# Walkability and its association with prevalent and incident diabetes among adults in different regions of Germany: results of pooled data from five German cohorts

**DOI:** 10.1186/s12902-019-0485-x

**Published:** 2020-01-13

**Authors:** Nadja Kartschmit, Robynne Sutcliffe, Mark Patrick Sheldon, Susanne Moebus, Karin Halina Greiser, Saskia Hartwig, Detlef Thürkow, Ulrike Stentzel, Neeltje van den Berg, Kathrin Wolf, Werner Maier, Annette Peters, Salman Ahmed, Corinna Köhnke, Rafael Mikolajczyk, Andreas Wienke, Alexander Kluttig, Gavin Rudge

**Affiliations:** 1 Institute of Medical Epidemiology, Biometrics and Informatics, Martin-Luther-University Halle-Wittenberg, Magdeburger Straße 8, 06112 Halle (Saale), Germany; 2 German Center for Diabetes Research (Deutsches Zentrum für Diabetesforschung DZD), Ingolstädter Landstraße 1, 85764 Neuherberg, Germany; 3 Centre for Urban Epidemiology, University Clinics Essen, Hufelandstr. 55, 45122 Essen, Germany; 4 Institute of Applied Health Research, University of Birmingham, Edgbaston, Birmingham B15 2TT UK; 5 German Cancer Research Center DKFZ (Deutsches Krebsforschungszentrum) Heidelberg, Im Neuenheimer Feld 280, 69120 Heidelberg, Germany; 6 Institute of Geosciences and Geography, Martin-Luther-University Halle-Wittenberg, 06099 Halle (Saale), Germany; 7 Institute for Community Medicine, University Medicine Greifswald, Ellernholzstr. 1-2, 17487 Greifswald, Germany; 8 Institute of Epidemiology, Helmholtz Zentrum München, Ingolstädter Landstr. 1, 85764 Neuherberg, Germany; 9 Institute of Health Economics and Health Care Management, Helmholtz Zentrum München, Ingolstädter Landstraße 1, 85764 Neuherberg, Germany; 10 Institute of Epidemiology and Social Medicine, University of Münster, Albert-Schweitzer-Campus 1, 48149 Münster, Germany

**Keywords:** Built environment, Walkability, Diabetes, Cardio-metabolic risk factors, Epidemiology

## Abstract

**Background:**

Highly walkable neighbourhoods may increase transport-related and leisure-time physical activity and thus decrease the risk for obesity and obesity-related diseases, such as type 2 diabetes (T2D).

**Methods:**

We investigated the association between walkability and prevalent/incident T2D in a pooled sample from five German cohorts. Three walkability measures were assigned to participant’s addresses: number of transit stations, points of interest, and impedance (restrictions to walking due to absence of intersections and physical barriers) within 640 m. We estimated associations between walkability and prevalent/incident T2D with modified Poisson regressions and adjusted for education, sex, age at baseline, and cohort.

**Results:**

Of the baseline 16,008 participants, 1256 participants had prevalent T2D. Participants free from T2D at baseline were followed over a mean of 9.2 years (SD: 3.5, minimum: 1.6, maximum: 14.8 years). Of these, 1032 participants developed T2D. The three walkability measures were not associated with T2D. The estimates pointed toward a zero effect or were within 7% relative risk increase per 1 standard deviation with 95% confidence intervals including 1.

**Conclusion:**

In the studied German settings, walkability differences might not explain differences in T2D.

## Background

Unhealthy diet and physical inactivity are important risk factors for developing non-communicable diseases, such as type 2 diabetes (T2D) [1]. While the prevention of such diseases is still focused on individual health behaviours, there is currently an increasing interest in setting-based prevention initiatives [2–4]. There is evidence that improved neighbourhood walkability, as characteristic of the built environment, increases walking and cycling. Hence, walkability may be associated with a reduced risk of obesity and T2D via increased transport-related and leisure-time physical activity [5–12].

Existing research showing a positive relationship between higher walkability and lower risk of incident and prevalent T2D comes mainly from Australia and North America [12, 13]. Since the built environment in Europe differs from Australia and North America [14, 15], it is not clear whether this association also exists in European countries. However, so far, there is only one study from Sweden showing no effect of walkability on incident T2D [16]. Our previous pooled analysis of data from five German cohorts indicated a weak association between higher walkability and lower body mass index (BMI) [17].

Most previous studies categorized continuous walkability measures, which is problematic in terms of loss of power and difficulties in pooling estimates from different studies [18]. Furthermore, most studies used a walkability score and did not assess walkability measures separately. A score does not permit conclusions as to which walkability parameter contributes most to the association with T2D and hampers comparability between studies since many options exist on which parameters to include in an index and how to weight them [19, 20].

In the current study, we assessed the association between three walkability measures and T2D prevalence and incidence in the German population using data from five German cohort studies.

## Methods

### Study population

Data from five population-based cohort studies from different German areas were included: The Heinz Nixdorf Recall Study (HNR), the Dortmund Health Study (DHS), both conducted in Western Germany, the Cooperative Health Research in the Region of Augsburg (KORA) S4 Survey from the South of Germany, The Cardiovascular Disease Living and Ageing in the city of Halle (CARLA) Study, and the Study of Health in Pomerania (SHIP), the latter two from the Eastern area of Germany. A detailed description of the studies can be found elsewhere [21–27]. Baseline data of all studies were collected between 1997 and 2006. Baseline response ranged from 56 to 69%. Except for the DHS cohort with only one follow-up examination, all other cohort studies conducted at least two follow-up examinations. The follow-up investigations took place between 2002 and 2016 with mean observation times ranging from 2.2 years to 13.6 years. Participation at follow-ups ranged between 53.5 and 76.6% (of all baseline participants).

The studies have been conducted according to the principles of the Declaration of Helsinki and have been approved by local ethics committees and written informed consent has been obtained.

**Fig. 1:**
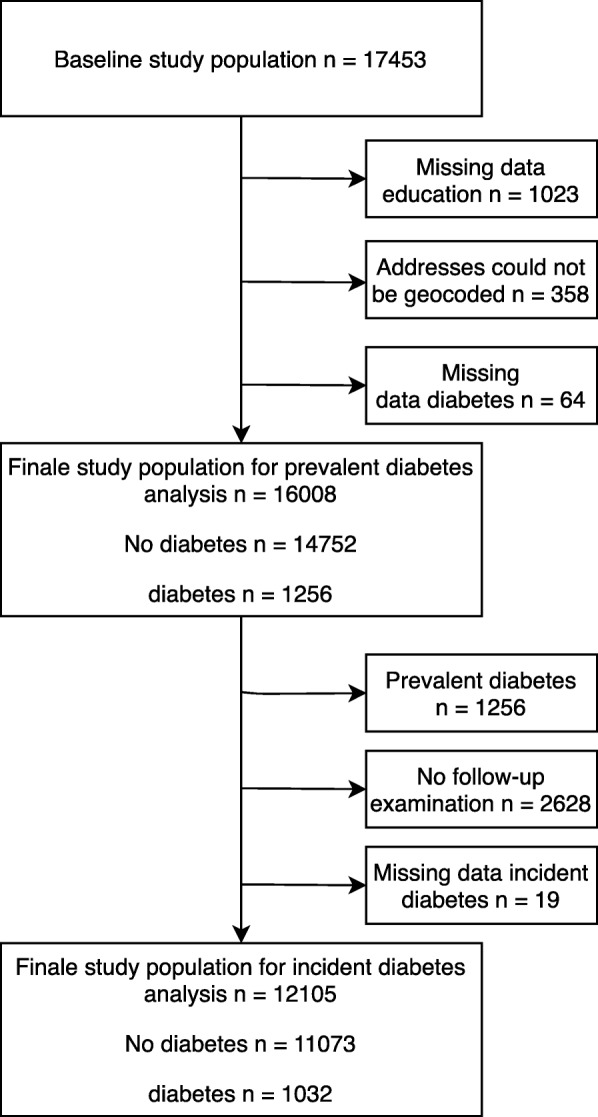
Study population for prevalent and incident diabetes analysis

In total, 17,453 participants were included in the pooled sample of the five cohort studies. Cross-sectional data from 16,008 and longitudinal data from 12,105 participants were available for analysing the association between the walkability measures and prevalent and incident T2D, respectively, after excluding participants with missing values for exposure, outcome, or covariates (Fig. 1).

### Walkability measures

**Fig. 2:**
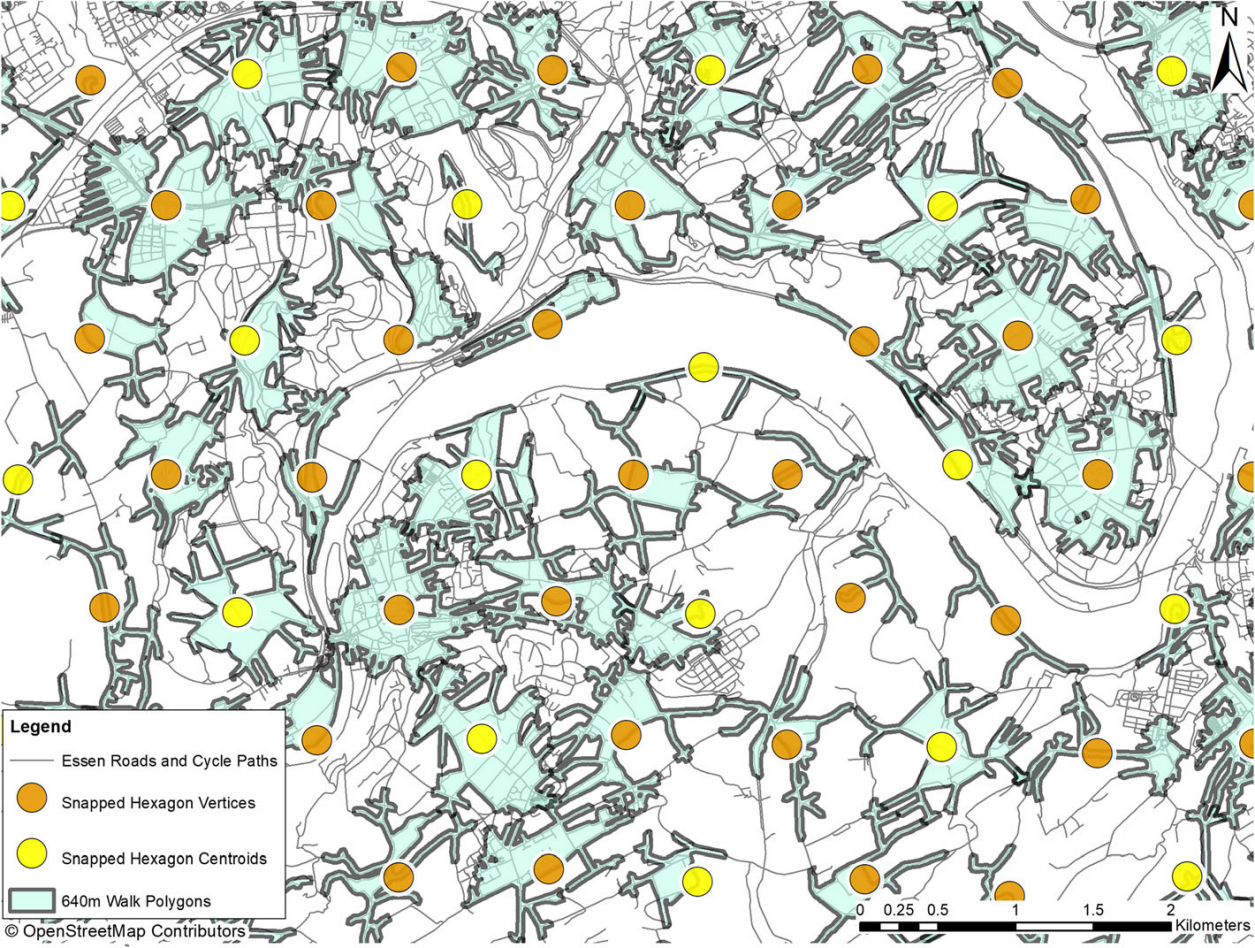
Walk polygons with hexagon centroids and vertrices. The figure shows one area of Essen city (Heinz Nixdorf Recall study area). For creating the map, OpenStreetMap data was used. OpenStreetMap® is open data, licensed under the Open Data Commons Open Database License by the OpenStreetMap Foundation. Note to the journal: please use colours for print

For deriving the walkability measures, the ArcGIS Geoinformation System in ESRI ArcMap Desktop versions 10.1 and 10.4 was used [Environmental Systems Research Institute (ESRI) 2012. 10.4, A.D.A.(ed.). Redlands]. We created a hexagonal sampling grid across each of the study regions covering the municipal boundary from which cohort participants had been recruited and a buffer of 1 kilometre beyond. Spatial interpolation will produce some spurious values at the edges of the areas it is applied to, so where possible it is performed on a larger area than needed and the resulting surface is trimmed to the extent of the study area. We picked 1000 m as the side length for the hexagons. The size of the hexagons was chosen pragmatically. We calculated hexagonal polygons depicting the area within a walking distance of 640 m for each of the hexagon’s vertices and centroids by using paths, walkways, and roads (Fig. 2).

The transport networks (here transit stations) and points of interest (POI) for the cities were provided by OpenSteetMap (OSM) in 2016 and processed using ArcGIS to create the network dataset.

The three walkability measures derived were the following:

POI: For each vertice and centroid of the hexagons we calculated a polygon representing the area that could be reached within a walking distance of 640 m, using roads, walkways and paths on the OSM network. There is very little research on what constitutes a short walk. The cut-off of 640 m was chosen because research carried out in London had proposed that the propensity to walk to access public transport declined rapidly after 640 m [28]. We took this a pragmatic definition of a short, accessible walking distance. We defined POI using OSM. These POI were geo-located and subsequently given a descriptive tag and allocated to a category. For example, an entry may be tagged as ‘bookshop’ in the category ‘shop’, a cash dispenser may be tagged ‘ATM’ in the category ‘amenity’. In each polygon we captured the number of points classified as a shop. In addition, we also selected some points classified as ‘amenity’ by undertaking a thorough review of the used tags. For each polygon, we thus summed up all shops and amenities tagged: ATM, bank, bar, Biergarten, café, fast food restaurant, pharmacy, pub, restaurant, and post office.

Transit stations: We followed exactly the same method to determine transit point availability as we did for POI. In the category ‘highway’ we captured all of the points tagged as ‘bus stop’. In the category ‘railway’ we captured all points tagged as ‘stop’, ‘tram stop’, ‘station’, ‘subway entrance’ ‘entrance’ or ‘platform’ and in the category ‘amenity’ we captured all the points tagged as ‘bus station’, and ‘ferry terminal’.

Impedance: Typically, connectivity is used as a proxy measure for impedance. Highly connected roads and paths will have many network notes (junctions of roads for example). Usually, the number of nodes of a road and path network in a given radius is counted to derive a simple metric of impedance. However, this approach does not capture physical barriers. Hence, we used an approach that would not only capture lack of intersections, but also physical barriers. From the centroid of each hexagon we calculated six journeys in six different directions: Northeast, Northwest, West, Southwest, Southeast, and East from the centre to each vertex of the hexagon. The six values vary according to ease of access in the various directions with higher values reflecting a lack of ease of walking in that direction. For example, if a point, which was 1000 m Euclidian distance away, was accessible by a journey of 2300 m this was 2.3 times larger than the Euclidian distance. A comparable 1000 m Euclidian distance that can be walked in 1050 m clearly has fewer barriers.

Each of the metrics was interpolated between the points to construct a surface. We used Kriging to interpolate values between the hexagonal points. Kriging estimates values between points of known values on a plane using a Gaussian regression process and is a widely used method in spatial modelling.

We intersected all created surfaces with the baseline residential addresses of the cohort’s participants. All walkability surfaces were created in 2016.

For DHS, only information on baseline residential addresses was available. Walkability measures could not be computed for 3% at baseline, 16% at follow-up 1 and 3.6% at follow-up 2, because addresses could not be geocoded.

## Outcomes

Prevalent and incident T2D were defined by self-report of physician-diagnosed diabetes or antidiabetic drug intake in the 7 days prior to the examination.

### Covariates

Number of years of education was derived from a standardized questionnaire. Years of education were classified based on the International Standard Classification of Education 1997 [29], including school years and years of vocational education in the total number of years with the categories: 9/10 years, 12/13 years, 14–17 years and 18 and more years. Eleven years is not included, since in Germany one can finish school after 9 or 10 years and then start vocational education or unskilled working, or one can finish after 12 or 13 years, which qualifies for university entry. Baseline BMI was derived from measured weight and height using comparable protocols in the five cohort studies. Self-reported hours per week of practicing sports were categorized with the following categories: more than 2 h, 1–2 h, less than 1 h of sports per week and practicing no sports.

### Statistical analysis

Sample characteristics were reported as means with standard deviation (SD) or medians with interquartile range (IQR) according to the distribution of the data or as frequencies (percentages) for categorical variables.

For associations between walkability measures and prevalent and incident T2D, we estimated risk ratios (RR) using modified Poisson regression with robust error variance [30, 31]. For better interpretability and comparability, we present estimates for z-standardized walkability measures.

In sensitivity analysis, we examined the association between walkability measures and T2D incidence in a sub-sample of participants whose addresses were the same during each of the follow-up assessments (hereafter ‘non-movers’). Additionally, we examined the association between walkability measures and T2D incidence in a sub-sample excluding all participants aged less than 30 years at baseline in order to exclude potential type 1 diabetes cases from the analysis. Finally, we also conducted an analysis in which we used the T2D status at the last follow-up as outcome in order to reduce the time gap between walkability assessment and T2D prevalence assessment.

We adjusted all models for sex, age at baseline, education, and cohort. Additionally, we examined if the associations differed in certain age groups (20–40 years, 41–60 years and over 60 years). Moreover, we adjusted the associations for practicing sports. All analyses were performed with SAS V.9.4 [32].

## Results

Of the baseline 16,008 participants, 1256 (7.8%) had prevalent T2D. During the follow up over a mean of 9.2 years (SD: 3.5, minimum: 1.6, maximum: 14.8 years), further 1032 participants developed T2D. Participants with prevalent or incident T2D were more often male, older, and had fewer years of education when compared to participants without T2D. Additionally, participants with T2D lived in areas with slightly more transit stations and POI when compared to participants without T2D. Impedance at the participant’s residential addresses was comparable in participants with and without T2D (Table 1). All estimates for the association between the walkability measures and T2D prevalence and incidence were within 7% of RR = 1 per 1 SD, with 95% confidence intervals including 1 (Table 2), showing no association between walkability measures and T2D. Given the large sample size, the 95% confidence intervals were narrow, indicating high precision of our estimates.

**Table 1:** Characteristics of participants

		Sample for examining prevalent T2D (*N* = 16,008)		Sample for examining incident T2D (*N* = 12,105)	
		Prevalent T2D at baseline (*N* = 1256)	No T2D at baseline (*N* = 14,752)	Incident T2D during follow-up (*N* = 1032)	No T2D during follow-up (*N* = 11,073)
Male	n (%)	707 (56.3)	7231 (49.0)	588 (57.0)	5271 (47.6)
Age at baseline	Mean (SD)	63.9 (9.8)	53.5 (13.9)	59.0 (9.9)	52.9 (13.4)
Education (years)					
9–10	n (%)	260 (20.7)	1598 (10.8)	112 (10.9)	1012 (9.1)
12–13	n (%)	613 (48.8)	7722 (52.3)	575 (55.7)	5731 (51.8)
14–17	n (%)	218 (17.4)	2786 (18.9)	189 (18.3)	2168 (19.6)
≥ 18	n (%)	165 (13.1)	2646 (17.9)	156 (15.1)	2162 (19.5)
Walkability measures					
Impedance	Mean (SD)	1623.9 (307.4)	1616.1 (289.9)	1615.3 (267.1)	1616.5 (288.6)
Transit stations	Median (Q1-Q3)	4.6 (2.7–6.9)	4.3 (2.3–6.9)	4.7 (2.5–7.1)	4.3 (2.4–6.9)
Points of interest	Median (Q1-Q3)	5.0 (2.3–10.1)	4.8 (2.1–9.5)	5.3 (2.6–9.8)	4.8 (2.2–9.0)
Risk factors for T2D					
Practicing sports					
More than 2 h/week	n (%)	196 (15.7)	3470 (23.7)	200 (19.5)	2804 (25.5)
1–2 h/week	n (%)	144 (11.5)	2767 (18.8)	156 (15.2)	2169 (19.7)
Less than 1 h/week	n (%)	97 (7.7)	1673 (11.4)	111 (10.8)	1257 (11.4)
No sports	n (%)	814 (65.1)	6758 (46.1)	560 (54.5)	4783 (43.4)
BMI at baseline	Mean (SD), n	30.5 (5.3), 1252	27.3 (4.6), 14,684	30.6 (4.8), 1024	27.0 (4.4), 11,042

*T2D* Type 2 diabetes, *SD* Standard deviation, *Q* Quartile, *BMI* Body mass index

**Table 2:** Association between T2D and walkability

					Sensitivity analysis			
	Prevalent T2D (*N* = 16,008)		Incident T2D (*N* = 12,105)		Incident T2D non-movers (*N* = 5901)		Incident T2D age ≥ 30 years at baseline (*N* = 11,416)	
	RR	95% CI	RR	95% CI	RR	95% CI	RR	95% CI
Impedance								
Crude	1.02	0.97, 1.08	1.00	0.94, 1.05	0.99	0.92, 1.07	1.00	0.95, 1.07
Adjusted	1.03	0.97, 1.09	1.00	0.94, 1.06	1.02	0.95, 1.11	0.99	0.93, 1.06
Transit stations								
Crude	1.05	0.99, 1.10	1.07	1.01, 1.13	1.09	1.00, 1.17	1.05	0.99, 1.11
Adjusted	1.03	0.97, 1.10	1.05	0.98, 1.13	1.05	0.97, 1.15	1.06	0.98, 1.14
Points of interest								
Crude	1.04	0.99, 1.09	1.06	0.99, 1.12	1.02	0.95, 1.10	1.05	0.99, 1.11
Adjusted	1.05	0.99, 1.11	1.01	0.95, 1.08	0.98	0.91, 1.07	1.01	0.95, 1.08

Adjusted models are controlled for sex, age at baseline, education, and cohort. *T2D* Type 2 diabetes, *RR* Relative risk, *CI* Confidence interval. RR are from modified Poisson regression models and reported per 1 standard deviation of walkability measures. RR over 1 indicate higher risk of T2D in areas with more transit stations and POI (indicators of better walkability). RR over 1 indicate higher risk of T2D in areas with higher impedance (indicator of poorer walkability)

For prevalent T2D analysis (N = 16,008): standard deviation (SD) impedance: 291.3, POI: 4.8, transit stations: 4.4

For incident T2D analysis (N = 12,105): SD impedance: 286.8, POI: 7.1, transit stations: 5.3

For incident T2D non-movers (N = 5901) SD impedance = 284.6, POI: 6.7, transit stations: 4.9

For incident T2D age ≥ 30 years at baseline (N = 11,416) SD impedance: 282.5, POI: 7.5, transit stations: 4.1

Results of a sensitivity analysis assessing the association between walkability and the most recent follow-up status on T2D were qualitatively the same (for impedance RR 0.99; 95% CI 0.95, 1.04; POI: 1.02; 0.98, 1.06; transit stations: 1.07; 1.01, 1.13, *n* = 9441).

These result of no association between walkability and T2D was confirmed by further analysis, were we stratified for age group and adjusted for practicing sports (See Additional file 1: Table S1 and S2).

## Discussion

In the present study we analysed data from 16,008 participants from five German cohort studies. Our results point towards a lack of association between walkability and T2D in the studied environments.

Walkability was measured in different ways in different studies, which hinders comparability of our results with the current literature. However, most studies showed a lower T2D risk with better walkability. Pooled effects in a recent review would translate into a 20% T2D risk reduction with better walkability [12].

Most studies that found associations between better walkability and decreased T2D risk used objective composite scores including measures we did not take into account, for example residential, population and intersection density, and land use mix [33–36]. While these studies combined different walkability measures into an index score and found association with diabetes, we aimed to analyze the contribution of single measures.

Christine and colleagues (2015) found associations for better subjective walkability measures, which we did not consider, and decreased T2D risk [37]. We focused on the classical and rather gross features of walkability that arose from urban planning. We did not consider fine features, such as bike path, pedestrian crossings, or avenues, nor did we consider green spaces and parks. Moreover, we did not include aesthetics and perceived safety. These walkability measures could be more important in determining especially leisure time related walking than single gross features of walkability [38]. Therefore, these measures would also be more important regarding T2D risks. Paquet et al. (2014) reported a 12% reduced risk with increasing walkability in a smaller sample and less years of follow-up when compared to our population [39]. The study took place in Adelaide, South Australia, which is different from European cities in terms of built environment attributes [14, 39].

The density and diversity of European cities and their city centres may have a greater potential to promote physical activity for transport and leisure time as when compared to Australia, where the structures of the cities are more car-oriented and more heterogeneous regarding walkability [38]. The homogeneity of the walkability measures in our study regions could explain the observed lack of association. Additionally, Paquet et al. (2014) analysed diabetes and prediabetes as one clinical endpoint, which hinders comparability with our results [39].

However, not all studies have found associations between walkability and T2D. Müller-Riemenschneider and colleagues (2013) reported that after adjustment for individual SES, the previously existing positive effect of walkability on incident T2D disappeared [40]. Nevertheless, the estimates still pointed towards a decreased T2D risk with better walkability.

The only other study we know about that was conducted in the European context found no association between walkability and diabetes in the city of Stockholm [16]. This study only included participants who were taking medication because of their disease. On the one hand, the exclusion of participants with T2D not taking medication could have underestimated the effect [41]. On the other hand, these results could also indicate the homogeneity of walkability measures in European cities, as indicated by our study.

Various specific factors could explain the null effect for T2D with more POI and transit stations in our study. First, POI included restaurants and fast food chains. Eating out of home is associated with obesity and could by increasing the T2D risk, diminish any positive effect of walkability [42]. Regarding transit stations, high cost of public transport, low frequency routes and transport that serves only few routes could hinder transport related walking and promote car-dependence, even though public transport is available. Consequently, this would result in a null effect, as observed in our study. Additionally, some environmental factors are associated with high urbanity and with high walkability. These factors, such as air pollution, could at the same time increase the risk of T2D and hence diminish the positive effect walkability has on T2D, which would result in no observable effect [43]. Regarding impedance, we did not observe any associations with T2D. This may be due to different ways of how impedance could work. People living in areas with high impedance could be less likely to walk, which would lead to lower activity and higher T2D risk. However, when it is inconvenient to use a car, activity could increase and T2D risk would decrease. Areas which have different road networks, parking availability and parking cost could be different in the effect impedance has on people’s walking and cycling behavior and hence on their T2D risk. A river as a geographical barrier could hinder transport related walking. At the same time, it could increase leisure time related walking, jogging or cycling for recreation.

In our recent cross-sectional analysis on a similar pooled study population, better walkability was associated with lower BMI, but the observed associations were rather weak [17]. The already weak positive effect of walkability via increased walking and cycling on BMI may simply not be strong enough to have any observable effects on T2D, which lies one step further down the causal chain. Additionally, when we stratified the associations by cohort, we observed that the association between better walkability and lower BMI was not consistent among the cohorts. As described above, even though walkability could contribute to increased walking and cycling behaviour and therefor to decreased BMI (even though to a very low extent), other factors related to walkability could diminish possible positive effects of walkability on health outcomes resulting from obesity and hence, resulting in a lack of association.

Some limitations need to be considered. First, diabetes was based on self-report. However, results of several studies indicate that for diabetes the validity of self-reports is generally high [44, 45]. Moreover, we could not adjust for residential self-selection and only adjusted for education as one part of individual SES, but not for income, occupation, or area level SES.

Participants, who choose to live in a walkable area, might be more health-conscious, have a higher income and live a healthier lifestyle than people, who cannot afford living in the city centre, where rents, but also connectivity as well as the amount of transit stations and POI might be higher. Hence, regardless of walking and cycling for recreation and transport, those people would have lower T2D risk than participants with low socioeconomic status, who are living in low walkable areas. Although we adjusted for education in our analysis, education alone does not reflect socioeconomic status, residential self-selection, and general health behaviour. Income level and social status influence T2D risk and walkability. However, we did not observe any association between walkability and T2D risk in crude and adjusted models and the adjustment for education only yielded minor changes in the association when compared to the crude association.

The strongest limitation is that the walkability measures were compiled for a much later time period then baseline data, which could have resulted in misclassification of walkability measures. However, we could show that the analysis based on the last follow-up status of T2D as outcome produced similar results. One can assume that if there is some fluctuation in for example points of interest over time, than still it occurs mostly within the same areas, minimizing the risk of misclassification. Furthermore, we did not include other important aspects of walkability, such as perceived aesthetics, safety, residential density, and presence of green spaces and parks. Lastly, there are some limitations of our walkability measures. Variety of POI was not explicitly taken into account and bus and tram stops might be very different in quality, according to high or low frequency routes. While impedance indicates lack of walkable streets, it can include rivers and forests which might be on the other hand highly attractive for walking.

Despite these limitations, the study has several strengths. Different regions and cities in Germany were taken into account. With pooling data from five cohorts, we were able to cover almost an entire European country. Most previous studies included single cities in a country and were mostly conducted in North America and Australia. This study is one of the first studies that examined the association between walkability and T2D in Europe.

## Conclusion

Overall, the results of our study rather indicate a lack of association between walkability and T2D risk in German settings. This might be due to the homogeneity of the walkability measures in the studied population.

## Supplementary information

**Additional file 1: Table S1.** Association between walkability and T2D by age group. **Table S2.** Association between walkability and T2D adjusted for practicing sports.

## Data Availability

The datasets generated and/or analysed during the current study are not publicly available due to data privacy but are available from the corresponding author on reasonable request.

## Abbreviations

BMI: Body Mass Index
CI: Confidence Interval
OSM: OpenStreetMap
POI: Points of interest
RR: Relative Risk
SD: Standard deviation
T2D: Type 2 Diabetes
