# Supplementary material for: Walkability and its association with prevalent and incident diabetes among adults in different regions of Germany: results of pooled data from five German cohorts
Source: BMC Endocr Disord. 2020 Jan 13;20:7. doi: 10.1186/s12902-019-0485-x (PMC6958624; doi:10.1186/s12902-019-0485-x)
Supplement: Supplementary file 1 — Additional file 1: Table S1. Association between walkability and T2D by age group. Table S2. Association between walkability and T2D adjusted for practicing sports. [file 12902_2019_485_MOESM1_ESM.pdf]

Additional file

Table 1. Association between walkability and T2D by age group.

	Prevalent T2D			Incident T2D		
	n	RR	95 % CI	n	RR	95 % CI
Impedance						
20 - 40 years	3013	1.01	0.71, 1.44	2286	1.20	0.97, 1.48
41 - 60 years	6900	0.92	0.83, 1.02	5709	0.94	0.85, 1.03
over 61 years	6095	1.07	1.00, 1.14	4110	1.02	0.94, 1.12
Transit stations						
20 - 40 years	3013	1.17	0.81, 1.69	2286	0.78	0.55, 1.11
41 - 60 years	6900	1.10	0.98, 1.23	5709	1.10	0.99, 1.22
over 60 years	6095	1.00	0.93, 1.09	4110	1.05	0.95, 1.16
Points of interest						
20 - 40 years	3013	1.15	0.82, 1.62	2286	0.85	0.66, 1.11
41 - 60 years	6900	1.15	1.05, 1.28	5709	1.03	0.94, 1.13
over 60 years	6095	1.01	0.94, 1.08	4110	1.02	0.94, 1.12

Adjusted models are controlled for sex, education, and cohort. T2D, type 2 diabetes; RR, relative risk; CI, confidence interval. RR are from modified Poisson regression models and reported per 1 standard deviation of walkability measures. RR over 1 indicate higher risk of T2D in areas with more transit stations and POI (indicators of better walkability). RR over 1 indicate higher risk of T2D in areas with higher impedance (indicator of poorer walkability).

Table 2. Association between walkability and T2D adjusted for practicing sports.

	Prevalent T2D (n=15919)		Incident T2D (n = 12040)	
	RR	95 % CI	RR	95 % CI
Impedance	1.02	0.97, 1.08	1.00	0.94, 1.07
Transit stations	1.03	0.96, 1.10	1.05	0.98, 1.13
POI	1.05	0.99, 1.11	1.01	0.95, 1.08

Adjusted models are controlled for sex, age at baseline, education, practicing sports, and cohort. T2D, type 2 diabetes; RR, relative risk; CI, confidence interval. RR are from modified Poisson regression models and reported per 1 standard deviation of walkability measures. RR over 1 indicate higher risk of T2D in areas with more transit stations and POI (indicators of better walkability). RR over 1 indicate higher risk of T2D in areas with higher impedance (indicator of poorer walkability).
